# Significance of CD44 expression in head and neck cancer: a systemic review and meta-analysis

**DOI:** 10.1186/1471-2407-14-15

**Published:** 2014-01-13

**Authors:** Jianqiang Chen, Jianding Zhou, Jie Lu, Hua Xiong, Xueli Shi, Liang Gong

**Affiliations:** 1 Department of Otorhinolaryngology, Affiliated Cixi Hospital of Wenzhou Medical, College, Cixi 315300, China

**Keywords:** Pan-CD44, CD44-v6, Head and neck cancer, Prognosis

## Abstract

**Background:**

CD44 has been reported to be involved with tumor growth and metastasis and has also been implicated as a CSC marker in head and neck squamous cell cancer (HNSCC). However, the prognostic value of CD44 still remains controversial; hence, we investigated the correlation between CD44 and the clinicopathological features of HNSCC by meta-analysis.

**Methods:**

A comprehensive search was performed using PubMed, ISI web of Science and China National Knowledge Infrastructure (CNKI) up to April 2013. Only studies with immunohistochemical staining of HNSCC were considered. Data on TNM classification, tumor grade, disease free survival and 3- or 5-year overall survival rate were extracted.

**Results:**

Thirty studies with 2102 patients met the inclusion criteria for the meta-analysis. Fifteen studies used anti-pan-CD44 antibody, 9 used anti-CD44-v6 antibody, 2 used anti-CD44-v3 and 2 used anti-CD44s antibody, 1 used anti-CD44-v9, and 1 used anti-CD44-v6,-v3 and -v4-5 simultaneously. The total percentage of CD44 expression was 57.8%, with 49.3% in oral cancer patients, 66.4% in pharynx and 54.7% in larynx cancer patients expressing CD44. No significant correlation between clinical features and CD44 expression was revealed for oral cancer patients, but CD44 was shown to be associated with advanced T categories (larynx: RR = 1.33, 95% CI 1.01-1.76; larynx & pharynx RR = 1.21, 95% CI 1.08-1.35), worse N categories (larynx: RR = 2.53, 95% CI 1.99-3.21; larynx & pharynx RR = 1.95, 95% CI 1.35-2.82), higher tumor grades (larynx & pharynx RR = 1.71, 95% CI 1.04-2.79) and 5-year OS rates (larynx: RR = 0.62, 95% CI 0.47-0.83; larynx & pharynx RR = 0.66, 95% CI 0.47-0.94) in patients with laryngeal and pharyngolaryngeal cancer. In stratified analysis, pan-CD44 and CD44-v6 expression were both correlated with 5-year OS rate of patients with laryngeal (CD44: RR = 0.66, 95% CI 0.46-0.95; CD44-v6 RR = 0.53, 95% CI 0.37-0.77) and pharyngolaryngeal cancer (CD44: RR = 0.56, 95% CI 0.34-0.93; CD44-v6 RR = 0.53, 95% CI 0.37-0.77).

**Conclusions:**

Our analysis suggested that CD44 is related to worse T category, N category, tumor grade and prognosis, in pharyngeal and laryngeal cancer, but no clear association was revealed between CD44 expression and oral cancer.

## Background

Although the treatment for head and neck cancer is improving rapidly, head and neck cancer is still the sixth most common cancer worldwide, mainly because it is usually difficult to diagnosis at an early stage [1]. The histopathological types and developmental origins of cancers in the head and neck (oral, pharynx and larynx) are highly homologous, and ninety percent of the tumors in the head and neck are squamous-cell carcinomas (HNSCCs), which present as aggressive and recurrent malignancies [2]. Therefore, understanding the precise biological behavior of HNSCC in the head and neck is very important for early diagnosis and outcome prediction.

Currently, the most accepted prognostic factors are TNM classification, which relies on the tumor size, and metastasis [1]. However, the TNM system cannot distinguish aggressive tumors from nonaggressive tumors of the same size. Therefore, it would be very beneficial to find one or more bio-markers for the prediction of the biological behavior of HNSCCs. Recently, a small population of cancer cells, referred to as cancer stem cells (CSCs), was revealed to account for tumor initiation, relapse and resistance to chemo- or radiotherapy; thus, eradicating CSCs is considered critical in cancer therapy [3,4]. The CSC hypothesis has also been coined for HNSCC in the head and neck; some cell surface markers have been reported as CSC markers in HNSCC cancers, such as CD44, CD133, ALDH1 and ABCG2 [5-7], and high expression of these markers is usually considered an indicator of poor prognosis. Among them, CD44 is the most reported CSC marker in HNSCC [8-10].

The CD44 receptor is a typeItransmembrane glycoprotein that was initially identified as a leukocyte antigen [11,12]. The alternative splicing of variable exons of CD44 results in abundant variants, which are denoted CD44v, and the isoform with no variable exons in the mRNA is named CD44 standard (CD44s) [13]. The smallest, standard isoform is CD44s, which is generally expressed on vertebrate cells, while CD44v is only expressed on some epithelial cells [12,13]. CD44 is the major hyaluronan (HA) receptor [14], and CD44 bound to HA has been proven to participate in various tumor biological activities, including tumor progression, metastasis and proliferation [15,16]. CD44 plays a critical role in cell migration, with involvement in multiple steps. Once activated, the cytoplasmic tail of CD44 interacts with the actin cytoskeleton, and CD44 is induced to the leading edge of migrating cells. Then, CD44 binds with CD62 on the endothelial cells, and thereafter, the migrating cells roll on the endothelial cells. This process is the initial step of cell migration called extravasation [12,17,18]. Although nearly all evidence has shown a negative role of CD44 in tumor progression, some conflicting reports have found a positive prognostic value of CD44 in head and neck cancers, especially in oral cancer [19-22], which indicates that some information regarding CD44 is still uncovered. Therefore, we present here a systemic review and meta-analysis of published studies on the association of CD44 expression with clinicopathological features in patients with head and neck cancer.

## Methods

### Literature search and eligibility criteria

A systematic literature search of the electronic database PubMed, ISI Web of Science and China National Knowledge Infrastructure (CNKI) up to April 2013 was performed. A random combination of the following terms was used for the search: ‘CD44’, ‘oral’, ‘larynx or laryngeal’, ‘pharynx, nasopharynx, oralpharynx, hypopharynx or pharyngeal’, and ‘tumor, neoplasm, cancer or carcinoma’. The titles and abstracts of potential references were manually examined to exclude irrelevant publications, and all of the remaining literature on the topic of interest was reviewed for additional pertinent studies.

The studies included in this meta-analysis were either randomized controlled studies (RCTs) or observational studies (case-control or cohort) that evaluated the relationship between CD44 expression and clinicopathological features or prognosis in head and neck cancer. Eligible studies met the following criteria: (a) proven diagnosis of HNSCC in head and neck (oral cavity, larynx or pharynx); (b) the CD44-positive group was defined by an immunohistochemistry method; (c) the correlation of CD44 with clinicopathological features and survival outcome (either disease free survival or overall survival) was analyzed; (d) HR/logHR and 95% confidence interval (CI)/standard error (SE) or crude data were provided; and (e) the articles were written in English or Chinese.

All information, including the titles and abstracts of the potential literature, were read by two reviewers (L.G and J.C) independently to exclude irrelevant publications. Then, the full texts of the extracted articles were carefully examined for comprehensive evaluation. Disagreements were resolved by discussion with a third reviewer (J.Z). Moreover, when multiple studies contained overlapping data, the one with the largest data set or newest data was included, and the others were excluded. Simultaneously, the references of extracted articles were also manually searched to avoid missing relevant studies. If the full text was unavailable, we contacted the authors for the data needed for the meta-analysis.

### Data extraction and quality assessment

All data from the eligible studies were extracted by two independent reviewers (J.Z and J.L) with a predefined table (Additional file 1: Table S1). Data tables were designed to extract all relevant data from texts, tables and figures, including author, year, country, patient number, detection method, duration of follow-up, T category, N category, distant metastasis, the antibody used in the article, positive rates of CD44 over-expression, disease free survival rate (DFS) and 3-/5- year overall survival (OS) rates. Because some articles showed survival data indirectly with a Kaplan-Meier curve, the software GetData Graph Digitizer 2.24 (http://getdata-graph-digitizer.com/) was applied to digitize and extract the data.

The cut-off score of the CD44 positive group varied among the different studies; we defined the CD44 positive group according to the original articles. Because several studies gave data on the 3-year survival outcome while others gave data on 5-year survival outcome, we analyzed both the 3-and 5-year overall survival rates here.

### Statistical analysis

Stata version 11 (StataCorp LP, TX) was used in this meta-analysis. The statistical process was performed according to the guidelines proposed by the Meta-Analysis of Observational Studies in Epidemiology group. Relative risk (RR) with a 95% confidence interval (95% CI) was calculated using Review Manager 4.2. The heterogeneity among the studies was measured using the Q test and I^2^ test. A fixed or random model was used depending on the heterogeneity analysis. The potential for publication bias was assessed by the Funnel plots and Egger’s regression test. P values < 0.05 were considered statistically significant. All P values above are two tailed. Subgroup analyses were performed to investigate the value of CD44 expression as a prognostic indicator for HNSCC patients in studies of different organs, geographical locations, sample sizes and follow-up durations. Among the variants of CD44, anti-pan-CD44 antibody and anti-CD44-v6 antibody were the most applied in the articles; therefore, we also analyzed all of the parameters stratified by pan-CD44 and CD44-v6. Sensitivity analyses were also performed by excluding each study individually. In addition, this meta-analysis was also addressed with standard PRISMA checklist and diagram (Additional files 2 and 3).

## Results

### Search results

**Figure 1:**
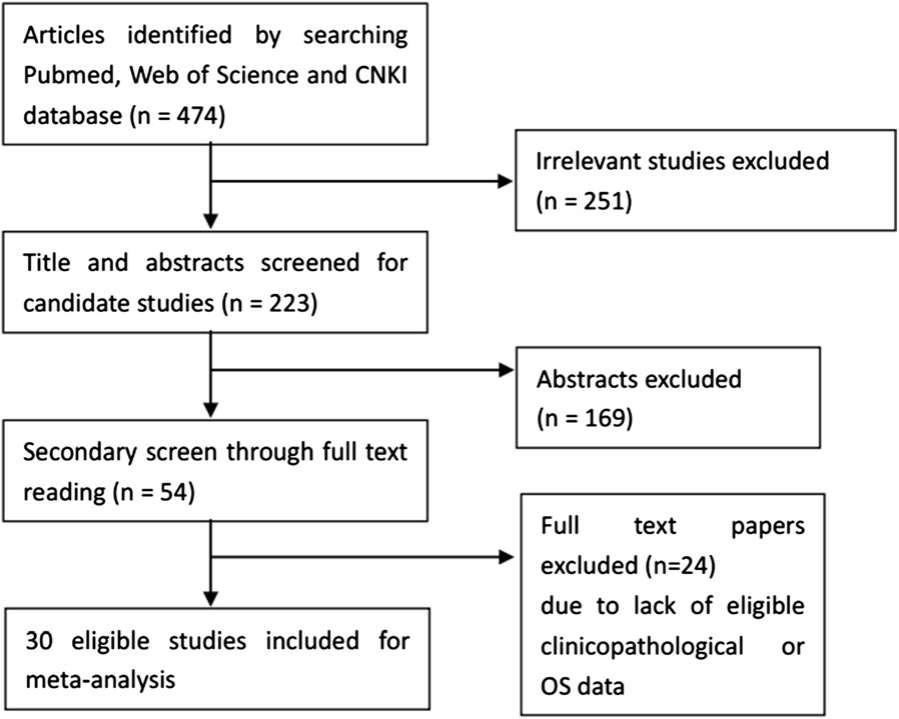
Flow chart for the selection of included articles.

Using the search strategy above, 474 articles were retrieved initially. After reviewing the titles and abstracts, 251 of those articles were excluded because they described non-human experiments or non-head and neck cancer-related studies or were non-original articles (reviews, letters). Of the remaining articles, 169 were excluded because they did not provide clinicopathological or survival rate data. Then, a secondary screen was performed that ruled out 24 studies without detailed data for analysis because, for example, the numbers of patients with high and low CD44 expression were not given. Eventually, a total of 30 studies were included in the present meta-analysis with approximately 2102 participants (Figure 1).

### Description of eligible studies

The characteristics of all included studies are listed in Table 1. Among the 30 studies analyzed, 20 were launched in Asia (China and Japan) and 10 in Europe or the USA. A total of 2102 patients with a median cohort size for each study of 58.5 (range 26 to 154) were included, and the mean follow-up time for the studies was 68.5 months (range 36 - 271 months). The TNM stage or tumor grade were reported in 24 studies, and patient outcome was reported in 15 studies (DFS and OS were presented in 5 and 12 articles, respectively). The median percentage of patients with CD44 positive expression was 57.8% (range 10.2 - 75.4%); for patients with oral cancer, pharynx cancer, and larynx cancer, the median percentages of patients with CD44 positive expression were 49.3% (range 10.2 - 70.9%), 66.4% (57.5 - 75.4%) and 54.7% (12 - 82.9%), respectively. All studies applied an immunochemistry staining method to detect CD44 expression: anti-pan-CD44 antibody was used in 15 studies [2,19-32], which detected all variations of CD44, anti-CD44-v6 antibody was used in 9 studies [33-41], anti-CD44-v3 antibody and anti-CD44s antibody were used together in 2 studies [42-45], anti-CD44-v9 was used in 1 study [46], and 1 study assessed CD44-v6,-v3 and -v4-5 simultaneously [47]. The most common cut-off values designating CD44 expression were cell membrane stainings of 50% (n = 16) and 25% (n = 5). Most of the included patients received surgical therapy, while 50 patients were treated with radiotherapy [21,42], 111 patients were given combined radiotherapy and surgery, and two patients received neoadjuvant chemotherapy [21,43].

**Table 1:** Characteristics of included studies

			**T category (T3,4 vs. T1,2)**		**N category (H vs. L)**		**M category (H vs. L)**		**Grade (G3 vs. G1,2)**		**DFS (death vs. survive)**		**OS 3 year (death vs. survive)**		**OS 5 year (death vs. survive)**
		N1	RR(95% CI)	N2	RR(95% CI)	N3	RR(95% CI)	N4	RR(95% CI)	N5	RR(95% CI)	N6	RR(95% CI)	N7	RR(95% CI)
**Over all**		13	1.15 (0.97, 1.35)	23	1.39 (1.07, 1.81)	4	1.79 (0.76, 4.26)	7	1.47 (1.00, 2.14)	5	1.50 (0.83, 2.71)	10	0.76 (0.59, 0.99)	12	0.76 (0.55, 1.05)
**Cancer type**															
**Oral**		2	0.75 (0.48, 1.16)	9	0.82 (0.54, 1.25)	2	1.99 (0.99, 3.99)	2	0.91 (0.50, 1.67)	4	1.86 (1.47, 2.37)	3	0.85 (0.49, 1.47)	5	0.91 (0.46, 1.78)
**Larynx**		8	1.33 (1.01, 1.76)	11	2.53 (1.99, 3.21)	-	-	4	2.13 (0.99, 4.58)	-	-	5	0.87 (0.62, 1.23)	5	0.62 (0.47, 0.83)
**Pharynx & Larynx**		11	1.21 (1.08, 1.35)	14	1.95 (1.35, 2.82)	2	1.77 (0.13, 23.59)	5	1.71 (1.04, 2.79)	1	0.53 (0.29, 0.97)	7	0.72 (0.52, 0.99)	7	0.66 (0.47, 0.94)
**Geographic area**															
**Asia**	Over all	9	1.39 (1.17, 1.64)	16	1.81 (1.26, 2.61)	3	1.82 (0.59, 5.63)	6	1.61 (1.01, 2.57)	2	0.98 (0.28, 3.4)	4	0.71 (0.56, 0.89)	4	0.8 (0.57, 1.14)
	Oral	-		3	0.75 (0.10, 5.62)	1	2.03 (0.99, 4.17)	1	0.72 (0.02, 4.33)	1	1.87 (0.9, 3.88)	-	-	-	-
	Larynx	6	1.60 (1.14, 2.25)	10	2.39 (1.65, 3.45)	-	-	4	2.13 (0.99, 4.58)	-	-	3	0.77 (0.66, 0.9)	3	0.69 (0.52, 0.91)
	Larynx & pharynx	9	1.39 (1.17, 1.64)	13	1.98 (1.33, 2.95)	2	1.77 (0.13, 23.59)	5	1.71 (1.04, 2.79)	1	0.53 (0.29, 0.97)	4	0.71 (0.56, 0.89)	4	0.8 (0.57, 0.94)
**Europe & USA**	Over all	3	0.88 (0.76, 1.02)	6	0.90 (0.68, 1.20)	1	1.46 (0.09, 22.93)	1	1.03 (0.56, 1.92)	2	3.08 (0.74, 12.78)	6	0.78 (0.48, 1.27)	8	0.64 (0.35, 1.16)
	Oral	1	0.57 (0.30, 1.11)	5	0.73 (0.52, 1.02)	1	1.46 (0.09, 22.93)	1	1.03 (0.56, 1.92)	2	3.08 (0.74, 12.78)	3	0.85 (0.49, 1.47)	5	0.90 (0.46, 1.78)
	Larynx	2	0.96 (0.85, 1.08)	1	1.85 (1.07, 3.18)	-	-	-	-	-	-	2	0.96 (0.24, 3.88)	2	0.46 (0.29, 0.72)
	Larynx & pharynx	2	0.96 (0.85, 1.08)	1	1.85 (1.07, 3.18)	-	-	-	-	-	-	3	0.61 (0.15, 2.5)	3	0.36(0.23, 0.57)
**Oral**	Pan-CD44	2	0.75 (0.48, 1.16)	3	0.58 (0.37, 0.92)	2	1.99 (0.99, 3.99)	2	0.91 (0.5, 1.67)	2	1.65 (1.68, 2.13)	3	0.85 (0.49, 1.47)	4	0.69 (0.32, 1.52)
	CD44-6	-	-	2	0.79 (0.29, 2.12)	-	-	-	-	-	-	-	-	-	-
**Larynx**	Pan-CD44	5	1.25 (0.90, 1.73)	6	2.83 (2.01, 3.98)	2	-	4	2.13 (0.99, 4.58)	-	-	5	0.87 (0.62, 1.23)	3	0.66 (0.46, 0.95)
	CD44-6	2	1.34 (0.99, 1.82)	4	1.92 (1.39, 2.64)	-	-	-	-	-	-	-	-	2	0.53 (0.37, 0.77)
**Larynx & pharynx**	Pan-CD44	6	1.23 (0.92, 1.65)	7	2.74 (1.98, 3.81)	1	6.65 (0.93, 47.63)	4	2.13 (0.99, 4.58)	1	0.53 (0.29, 0.97)	6	0.794 (0.56, 1.1)	4	0.56 (0.34, 0.93)
	CD44-6	3	1.15 (0.90, 1.48)	5	1.47 (0.90, 2.42)	1	1.03 (0.94, 1.13)	-	-	-	-	-	-	2	0.53 (0.37, 0.77)
**Sample size**^**a**^	<58.5	4	1.79 (0.82, 3.89)	10	1.388 (0.83, 2.33)	1	6.65 (0.93, 47.63)	3	1.34 (0.5, 3.58)	3	1.71 (0.44, 6.69)	3	0.34 (0.21, 0.54)	6	0.55 (0.22, 1.37)
	≥58.5	9	1.03 (0.91, 1.17)	13	1.40 (1.01, 1.95)	3	1.62 (0.87, 3.03)	4	1.62 (0.91, 2.88)	2	1.65 (1.28, 2.13)	7	0.91 (0.69, 1.2)	6	0.80 (0.57, 1.13)
**Follow time(month)**^**b**^	<68.5	7	1.04 (0.89, 1.20)	8	1.29 (0.80, 2.10)	2	4.51 (0.96, 21.13)	5	1.42 (0.96, 2.08)	5	1.50 (0.83, 2.71)	7	0.88 (0.66, 1.17)	8	0.89 (0.62, 1.28)
	≥68.5	6	1.37 (0.94, 2.01)	15	1.45 (1.03, 2.05)	2	1.28 (0.36, 4.50)	2	1.27 (0.11,14.01)	-	-	3	0.39 (0.25, 0.62)	4	0.43 (0.29, 0.65)

a: median of sample size for each study.

b: median of follow-up time for each study.

H: high expression; L: low expression; DFS: disease free survival; OS: overall survival.

### Relationship of CD44 with clinical features and patient survival

**Figure 2:**
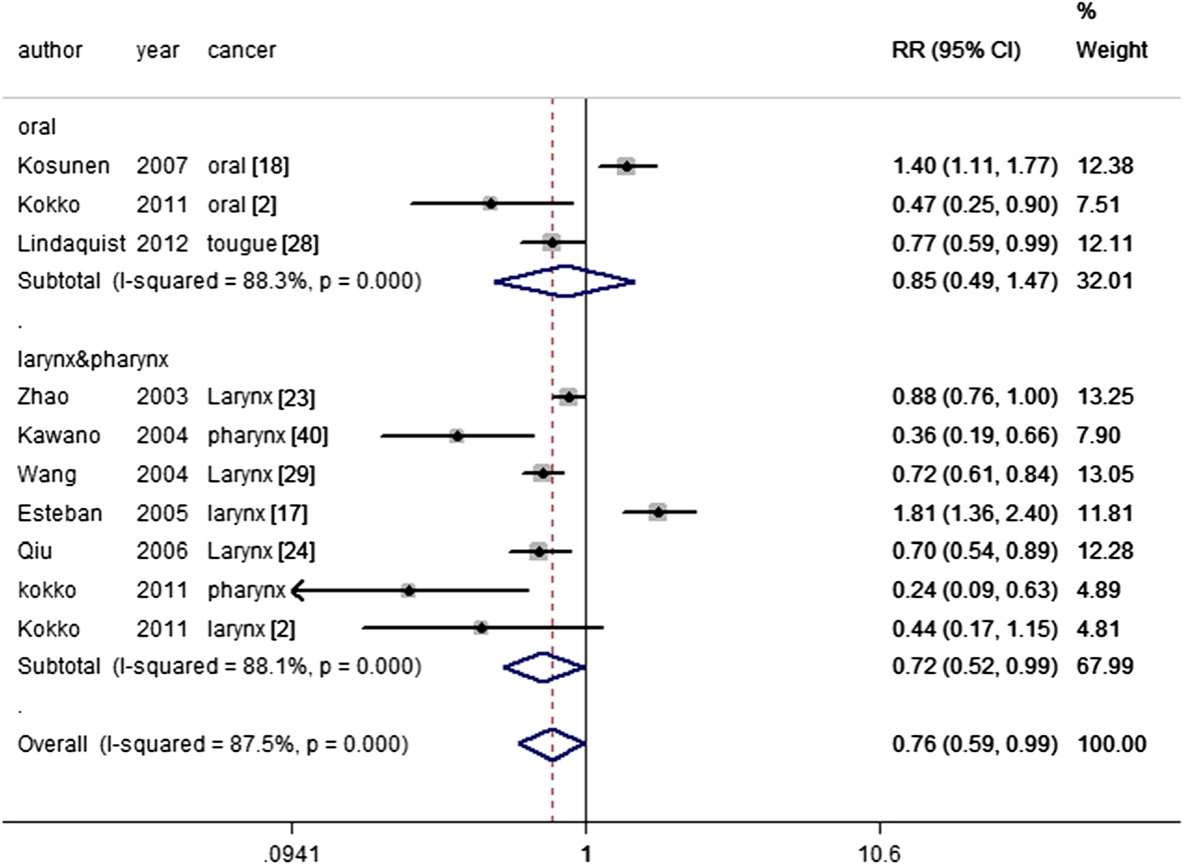
CD44 expression and 5-year OS rate stratified to oral and pharyngolaryngeal cancer.

**Figure 3:**
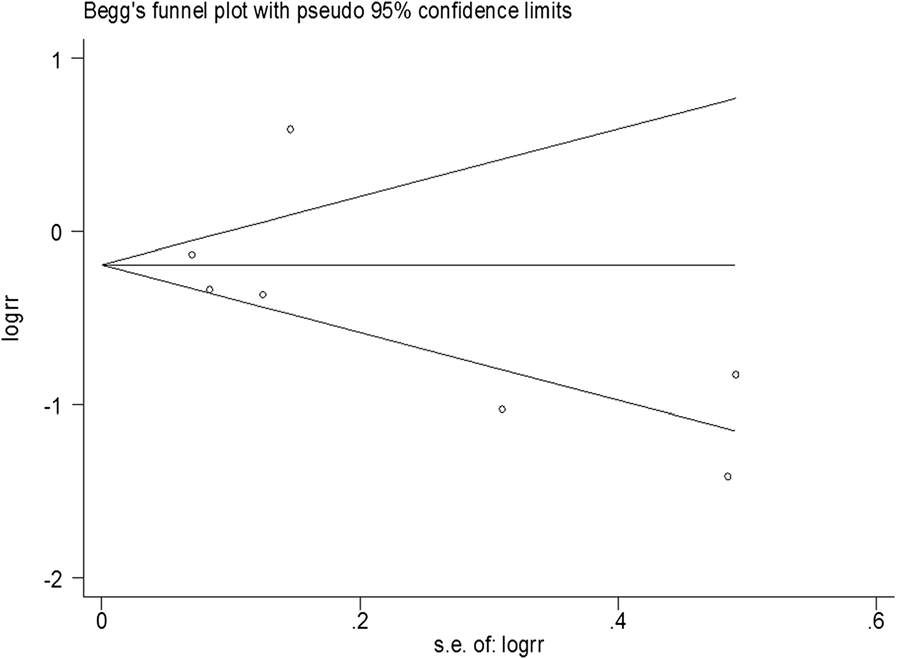
Begg’s test results of pharyngolaryngeal cancer patients' 5-year OS rate.

Additional file 4: Table S2 shows the summary RR of the clinicopathological features in patients with CD44 high and low expression. The pool analysis found a minimally significant association between CD44 and clinical features; the N grade (RR = 1.39, 95% CI 1.07-1.81) and 3-year OS rate (RR = 0.76, 95% CI 0.59-0.99) were negatively correlated with CD44 expression. Egger's test did not show any publication bias for the above data (Additional file 1: Table S1). Because CD44 expression may be organ specific, we performed a stratified analysis of oral, larynx and pharyngolaryngeal cancer. Although no significant relationship was found between clinical features and CD44 expression in oral cancer, CD44 expression was shown to be associated with worse T categories (larynx: RR = 1.33, 95% CI 1.01-1.76; larynx & pharynx RR = 1.21, 95% CI 1.08-1.35; Additional file 5: Figure S1), worse N categories (larynx: RR = 2.53, 95% CI 1.99-3.21; larynx & pharynx RR = 1.95, 95% CI 1.35-2.82; Additional file 6: Figure S2), higher tumor grades (larynx & pharynx RR = 1.71, 95% CI 1.04-2.79; Additional file 7: Figure S3), worse 3-year survival rates (larynx & pharynx RR = 0.72, 95% CI 0.52-0.99) and worse 5-year survival rates (larynx: RR = 0.62, 95% CI 0.47 - 0.83; larynx & pharynx RR = 0.66, 95% CI 0.47 - 0.94; Figure 2) in laryngeal and pharyngolaryngeal cancer. Here, Egger's test showed that a publication bias was present for the T category (larynx: *p* = 0.033; larynx & pharynx: *p* = 0.033) and tumor grade (larynx & pharynx: *p* = 0.01), but there was no evidence of publication bias for the 3-year (larynx & pharynx: Egger's test *p* = 0.454; Begg's test *p* = 0.76) and 5-year overall survival rates (larynx: Egger's test *p* = 0.137 Begg's test *p* = 1; larynx & pharynx: Egger's test *p* = 0.355 Begg's test *p* = 0.548 Figure 3). Sensitivity analyses also revealed the stability of our results (Additional file 8: Figure S4), indicating that CD44 expression was a prognostic factor for laryngeal and pharyngeal HNSCC patients .

### Subgroup analyses

Additional file 4: Table S2 presents the detailed results of the subgroup analyses. In addition to the analysis of expression in specific organs, which was presented above, we also evaluated the data of studies in different geographic locations and the data of studies using different antibodies. The results revealed that CD44 expression predicted worse 5-year OS in HNSCC patients of the pharynx and larynx and that this result was similar for the studies in Asia (RR = 0.8, 95% CI 0.57 - 0.94) and Europe/USA (RR = 0.36, 95% CI 0.23 - 0.57). However, CD44 expression was not found to be a significant predictor of 3-year OS in European patients (RR = 0.61, 95% CI 0.15 - 2.5) as one Spanish study showed the opposite result from the other European studies [20]. In consideration of the clinical features, CD44 expression was also associated with larger tumor size (RR = 1.39, 95% CI 1.17 - 1.64) and lymph node metastasis (RR = 1.98, 95% CI 1.33 - 2.95) in pharyngolaryngeal cancer of Asian patients; however, this phenomenon was not found in European patients, which may due to the limited number of European articles. Moreover, CD44 expression did not show significant correlation with OS rate or any clinical characteristics in Asian or European oral cancer patients. Because CD44 has many variants that have been reported with distinct biological functions, we divided the studies into the anti-pan-CD44 and anti-CD44-v6 subgroups, which were the major antibodies applied in these studies, for each organ. In oral cancer, pan-CD44 expression was not associated with OS rate, but high CD44 level did predict a better DFS rate (RR = 1.65, 95% CI 1.68 - 2.13) [21,42]. Furthermore, a lower risk of lymph node metastasis was related with pan-CD44 expression in oral cancer patients (RR = 0.58, 95% CI 0.37 - 0.92), but CD44-v6 expression did not reveal a similar correlation (RR = 0.79, 95% CI 0.29 - 2.12). On the other hand, pan-CD44 and CD44-v6 expression were found to be related to higher N grade in laryngeal cancer patients (pan-CD44, RR = 2.83, 95% CI 2.01 - 3.98; CD44-v6 RR = 1.92 95% CI 1.39-2.64), but neither pan-CD44 nor CD44-v6 expression showed a relationship with T grade or tumor grade in laryngeal or pharyngolaryngeal cancer patients (Additional file 4: Table S2). Furthermore, a poorer 5-year OS rate was associated with pan-CD44 and CD44-v6 expression both in laryngeal (CD44: RR = 0.66, 95% CI 0.46 - 0.95; CD44-v6 RR = 0.53, 95% CI 0.37-0.77) and pharyngolaryngeal cancer (CD44: RR = 0.56, 95% CI 0.34 - 0.93; CD44-v6 RR = 0.53, 95% CI 0.37-0.77). However, neither CD44 nor CD44-v6 was correlated with the 3-year OS rate in patients with laryngeal cancer, which implies that a longer follow-up time is needed for head and neck patients.

## Discussion

CD44 is one of the most frequently observed cancer stem cell (CSC) markers in solid tumors, and it was revealed to be a target of the Wnt pathway [48], which is accepted as a main pathway for the stemness maintenance of CSCs, and usually accepted as a poor prognosis marker. However, most reports that used CD44 as a CSC marker used the pan-CD44 antibody, which recognizes all variants of CD44; thus, these studies provide limited knowledge about the relationship between specific CD44 variants and CSCs. Very recently, several studies found a distinct role between CD44v and CD44s. The EMT process in several epithelial cells was accompanied by a transition in CD44 isoforms from CD44v to CD44s, and CD44s has been shown to promote the EMT process [49,50]. However, hypoxia and hypoxia-inducible factor-1α have been shown to stimulate CD44v expression, sialyl Lewis X glycan has been found to attach to CD44v rather than to CD44s, and CD44v has been shown to be a ligand for E-selectin during tumor metastasis [51]. These findings indicate complicated roles of CD44s and CD44v in tumor progression. The expression of CD44 standard, v3, v5, v6, and v9 have been reported in HNSCC patients [36,37,46,52,53]. Of interest, we found that high CD44 expression in HNSCC patients indicated different clinical values in the oral cavity and pharynx or larynx. CD44 high expression strongly predict poorer T grade, N grade and worse overall survival rate in the larynx and pharynx, but better disease free survival rate and no association with any clinicopathological features in oral cancer. According to our systemic review results, CD44 expression may have a different prognostic value in the squamous cancer of the oral cavity and pharynx or larynx. To the best of our knowledge, there is still no direct explanation for this observation, so here, we mainly focused on the unexpected meta-analysis result and tried to give a rational explanation by reviewing the literature.

Although many of these works showed a negative relationship between CD44 protein level and prognosis in oral cancer patients, several reports found that an increased transcriptional level of CD44 indicated disease progression. Rajarajan et al. [54] found that the mRNA level of CD44 was significantly increased in oral squamous cell carcinomas; in addition, Lin et al. [55] revealed the CD44 mRNA levels in the peripheral blood of patients with locally advanced oral or oropharynx cancer were much higher than in healthy people, and a high CD44 mRNA level was significantly related to poor prognosis. This interesting phenomenon prompted us to review the antibodies applied by all of the studies in this meta-analysis. As different CD44 isoforms have distinct extracellular domains while retaining the same transmembrane and intracellular domain structure, nearly all works in this review used antibodies that targeted the extracellular domain of CD44. It is important to note that decreased intensity of CD44 by extracellular domain targeted antibody detection does not definitively indicate low CD44 expression because the shedding of CD44, which means the cutting off of the extracellular domain of CD44, could cause the false appearance of decreased CD44 expression. In fact, CD44 shedding has been observed in many human tumors, including breast, lung, colon and ovarian carcinomas [56], and indeed takes part in tumorigenesis and tumor metastasis. In the late phase of HA-CD44 binding, the CD44 at the leading edge of the cell is cleaved by a disintegrin and metalloproteinase domain (ADAM) protein and matrix metalloproteinase 14 (MMP14), and this cleavage is required for tumor cell migration [57]. Furthermore, researchers have found that the CD44 ectodomain cleavage subsequently induces the CD44 intramembranous cleavage, and the intramembranous cleavage generates the CD44 intracellular domain, which is translocated to the nucleus as a signal transduction molecule and activates the transcription of various genes, including CD44 [58,59]. This positive feedback loop supports our hypothesis that an increase in CD44 expression during oral cancer progression could be covered by its shedding.

The shedding of CD44 has been reported to be mainly associated with ADAM10 and ADAM17. ADAM10 is co-localized with CD44, and inhibition of ADAM10 could decrease the shedding of CD44 [60]. In oral cancer, it was shown that ADAM10 and ADAM17 expression were positively related to CD44 cleavage status; furthermore, both of them were more highly expressed in advanced oral cancer and were indicators of nodal metastasis [61-63]. However, little research on ADAM 10/17 could be found for pharyngolaryngeal cancer; therefore, we referred to the Oncomine database (http://www.oncomine.org). The results showed that, according to gene microarray analysis, ADAM10 was not increased in hypopharyngeal cancer compared to normal tissue [64] and that both ADAM10 and ADAM17 were not presented in the list of genes with significantly altered expression levels in pharyngolaryngeal cancer compared to normal tissue. Although these results were based on mRNA levels, they could also strongly indicate a difference in CD44 proteolytic enzyme expression between oral and pharyngolaryngeal cancer and may explain the different role of CD44 in the oral cavity and pharynx. With this in mind, we propose that future studies should evaluate the cleavage of CD44 rather than intact CD44 protein for prognosis prediction.

According to the analysis above, CD44 indeed participates in oral cancer progression, but cell staining of CD44 may not be accurate enough to reflect the authentic levels of CD44 because of the shedding of CD44. CD44 cleavage results in the extracellular epitope of CD44 being detached from the cell membrane and existing in its soluble form (sCD44). Although CD44 was widely expressed in normal oral mucosa, CD44 cleavage was found to occur at a low level or not at all in normal oral mucosa [65] and to be overexpressed in oral cancer tissue [61], which could be explained by the proteolytic effect of ADAM. Additionally, it was reported that the salivary sCD44 levels were much higher in HNSCC patients than in patients with benign disease [66,67]; however, the plasma level of sCD44 was not significantly different between oral cancer patients and normal controls, which may be attributed to the relatively small burden of oral tumors that may not alter the basal level of CD44 [66]. The same situation has been reported in gastrointestinal stromal tumors (GIST); CD44 cleavage activity was shown in 87.1% of GISTs but was absent in normal tissues, and increased CD44 cleavage was significantly related to advanced TNM stage and poor prognosis [68]. Here, we proposed that detecting the CD44 cleavage or soluble CD44 levels in tissue sections or oral rinses would be better than cell staining for prognostic prediction; another advantage of oral rinses is their convenience and noninvasiveness. Furthermore, based on the above discussion, CD44 may have limited utility in identifying oral CSCs [69], but some other cell surface markers have been reported to be expressed on stem-like cells in oral cancer, such as CD133, ABCG2 and ALDH1 [5,6]. In contrast, high CD44 expression was proven to be closely related to poor prognosis and was clearly enriched in stem-like cells in pharyngeal or laryngeal cancers [70-72].

Although we performed a comprehensive analysis of the association between CD44 expression and patient clinicopathological parameters for HNSCC, there were some limitations to this meta-analysis. First, the confounders were hard to control in the case-control studies, which may have affected the authentic prognostic value of CD44 expression. Second, except for CD44-v6, our systematic review and meta-analysis could not clarify the association between other CD44 variants and patient survival because of insufficient eligible information. Third, high quality studies with complete reports, including TNM stage and survival data, were limited, which may compromise our conclusions. In addition, there could be potential language bias in this meta-analysis because we only considered the literature written in English and Chinese. In conclusion, our systematic review and meta-analysis suggested that cell staining of CD44 has different prediction values for oral and pharyngolaryngeal cancer. In pharyngolaryngeal cancer, high CD44 staining intensity indicated worse T grade, worse N grade and a markedly shortened OS; meanwhile, cell staining of CD44 is not recommended for any clinicopathological or survival prediction for oral squamous cancer patients based on our review. The sCD44 evaluation may be a good substitute in oral cancer patients, and additional well-designed studies are needed to draw an authentic conclusion.

## Conclusion

Our analysis indicated prognostic value of CD44 expression were different between laryngopharyngeal cancer and oral cancer, that CD44 related to worse T category, N category, tumor grade and prognosis in pharyngeal and laryngeal cancer, but no significant association was revealed between CD44 and oral cancer.

## Abbreviations

HNSCC: Head and neck squamous cell carcinoma; CI: Confidence interval; CSC: Cancer stem cell; IHC: Immunohistochemistry; OR: Odds ratio; OS: Overall survival; RR: Relative risk.

## Competing interests

The authors declare that they have no competing interests.

## Authors’ contributions

LG and JC participated in extracting the data and wrote the manuscript. JZ and JL performed the statistical analysis. HX acbvnd XS carried out literature search and data collection. All authors approved the final manuscript.

## Pre-publication history

The pre-publication history for this paper can be accessed here:

http://www.biomedcentral.com/1471-2407/14/15/prepub

## Supplementary Material

Additional file 1: Table S1 Heterogeneity test and publication bias analyses among studies. Click here for file

Additional file 2 PRISMA 2009 Checklist. Click here for file

Additional file 3 PRISMA 2009 Flow Diagram. Click here for file

Additional file 4: Table S2 Heterogeneity test and publication bias analyses among studies included. Click here for file

Additional file 5: Figure S1 CD44 expression and T category stratified to oral and pharyngolaryngeal cancer. Click here for file

Additional file 6: Figure S2 CD44 expression and N category stratified to oral and pharyngolaryngeal cancer. Click here for file

Additional file 7: Figure S3 CD44 expression and tumor grade stratified to oral and pharyngolaryngeal cancer. Click here for file

Additional file 8: Figure S4 Sensitivity analysis of 5-year OS rate. Click here for file
