# Supplementary material for: Significance of CD44 expression in head and neck cancer: a systemic review and meta-analysis
Source: BMC Cancer. 2014 Jan 13;14:15. doi: 10.1186/1471-2407-14-15 (PMC3893437; doi:10.1186/1471-2407-14-15)
Supplement: Additional file 1: Table S1 — Heterogeneity test and publication bias analyses among studies. [file 1471-2407-14-15-S1.docx]

Additional file 1: Table S1

| **No** | **year** | | **Author** | **country** | **organ** | **Number** | **follow-up time** | **cut-off score(H/L)** | **CD44 antibody type** | **T (1,2/3,4)** | **N(P/N)** | **M(P/N))** | **Grade (1,2/3)** | **DFS**  **(relapse/free)**  **5-year** | **OS- 3year**  **(dead/ alive)** | **OS 5-year**  **(dead/ alive)** |
| --- | --- | --- | --- | --- | --- | --- | --- | --- | --- | --- | --- | --- | --- | --- | --- | --- |
| **1** | 1998 | Mori[[1](#_ENREF_1)] | | Japan | Oral | 86 | NA | 50%(54/32) | pan-cd44 | NA | NA | H(24/30);L(7/25) | NA | NA | NA | NA |
| **2** | 1998 | Ue[[2](#_ENREF_2)] | | Japan | Oral | 40 | NA | 50%(9/31) | cd44-v9 | NA | H(6/3); L(5/23) | NA | H(3/6); L(11/20) | NA | NA | NA |
| **3** | 1999 | Song[[3](#_ENREF_3)] | | China | Larynx | 69 | NA | membrane staining(36/33) | cd44-v6 | NA | H(18/18); L(6/27) | NA | H(19/17); L(22/11) | NA | NA | NA |
| **4** | 2000 | Musuda[[4](#_ENREF_4)] | | Japan | Tougue | 38 | median 60 months | 65%(25/13) | CD44s | NA | NA | NA | NA | H(7/18); L(8/5) | NA | NA |
| **5** | 2000 | Wang[[5](#_ENREF_5)] | | China | Larynx | 46 | NA | 30%(21/25) | pan-cd44 | H(3/18); L(17/8) | H(14/7); L(4/21) | NA | H(13/8); L(22/3) | NA | NA | NA |
| **6** | 2001 | Xiao[[6](#_ENREF_6)] | | China | Oral | 26 | NA | 50%(6/20) | pan-cd44 | NA | H(0/6); L(11/9) | NA | H(6/0); L(15/5) | NA | NA | NA |
| **7** | 2001 | Fonseca[[7](#_ENREF_7)] | | Portugal | Oral | 56 | NA | 50%(21/35) | cd44-v3 | NA | H(9/26); L(10/11) | NA | H(29/6); L(17/4) | NA | NA | NA |
|  | 2001 | Fonseca | | Portugal | Oral | 56 | NA | 50%(37/19) | cd44-v6 | NA | H(12/15); L(7/12) | NA | H(28/9); L(18/1) | NA | NA | NA |
|  | 2001 | Fonseca | | Portugal | Oral | 56 | NA | 50%(18/38) | cd44-v4-5 | NA | H(5/13); L(14/24) | NA | H(13/5); L(33/5) | NA | NA | NA |
| **8** | 2002 | Bankfalvi[[8](#_ENREF_8)] | | Germany | Oral | 46 | median 63 months | 75%(18/26) | cd44-v3 | NA | H(6/12); L(10/16) | NA | NA | H(8/10); L(24/2) | H(5/13); L(19/7) | H(6/12); L(19/7) |
| **9** | 2003 | Gonzalez-Moles[[9](#_ENREF_9)] | | Spain | Tougue | 56 | median 60 months | 50%(40/16) | pan-cd44 | NA | NA | NA | NA | NA | NA | H(14/2); L(5/35) |
| **10** | 2003 | Zhao[[10](#_ENREF_10)] | | China | Larynx | 110 | 5 year | 25%(78/32) | pan-cd44 | NA | H(28/50); L(4/28) | NA | H(34/44); L(24/8) | NA | H(14/64); L(2/30) | H(20/58); L(3/29) |
| **11** | 2003 | XU[[11](#_ENREF_11)] | | China | Larynx And Pharynx | 154 | 10 years | 80%(122/32) | cd44-v6 | H(55/67);L(15/17) | H(66/56); L(23/9) | H(4/118);L(2/30) | NA | NA | NA | NA |
| **12** | 2004 | Kawano[[12](#_ENREF_12)] | | Japan | Pharyx | 57 | 5-129 months | 5%(43/14) | CD44s | H(1/42);L(2/12) | H(39/4); L(12/2) | NA | H(2/41); L(2/12) | NA | H(32/11); L(4/10) | H(35/8); L(7/7) |
| **13** | 2004 | Wang[[13](#_ENREF_13)] | | China | Larynx | 106 | 5 year | 25%(72/34) | pan-cd44 | H(37/35);L(19/15) | H(23/49); L(2/32) | NA | H(19/53); L(11/23) | NA | H(22/50); L(1/33) | H(35/37); L(4/30) |
| **14** | 2005 | Esteban[[14](#_ENREF_14)] | | Spain | Larynx | 137 | median 36 months | 50%(90/47) | pan-cd44 | H(15/75);L(7/39) | NA | NA | NA | NA | H(7/83); L(23/24) | NA |
| **15** | 2006 | Qiu[[15](#_ENREF_15)] | | China | Larynx | 60 | 3 year | 25%(41/19) | pan-cd44 | H(15/26);L(13/6) | H(11/30); L(0/19) | NA | H(9/32); L(15/4) | NA | H(14/27); L(1/18) | NA |
| **16** | 2006 | Xie[[16](#_ENREF_16)] | | China | Oral | 50 | NA | 50%(26/24) | cd44-v6 | NA | NA | NA | H(24.2); L(12/12) | NA | NA | NA |
| **17** | 2007 | Kosunen[[17](#_ENREF_17)] | | Finland | Oral | 138 | 0.59-271 months median 53 months | 50%(56/82) | pan-cd44 | H(48/10);L(56/24) | H(9/49); L(22/58) | H(1/55);L(1/81) | H(43/15); L(45/37) | H(11/45);L(45/37) | H(12/44); L(36/46) | H(13/43); L(42/40) |
| **18** | 2007 | staibano[[18](#_ENREF_18)] | | Italy | Larynx | 51 | 96 months | 66.7(23/28) | cd44-v6 | NA | NA | NA | NA | NA | H(11/12); L(2/26) | H(13/10); L(3/25) |
| **19** | 2007 | Huang[[19](#_ENREF_19)] | | China | larynx | 46 | NA | 5%(33/13) | cd44-v6 | NA | H(22/11); L(4/9) | NA | H(22/11); L(10/3) | NA | NA | NA |
| **20** | 2008 | Lu [[20](#_ENREF_20)] | | China | larynx | 64 | NA | 50%(9/55) | cd44-v6 | H(2/6);L(26/29) | H(7/2); L(31/24) | NA | NA | NA | NA | NA |
| **21** | 2009 | Gao[[21](#_ENREF_21)] | | China | larynx | 75 | NA | 50%(9/66) | cd44-v6 | H(2/7);L(26/40) | H(7/2); L(31/35) | NA |  | NA | NA | NA |
| **22** | 2009 | Guo[[22](#_ENREF_22)] | | China | larynx | 70 | 69.6 months | 50%(58/12) | cd44-v6 | NA | NA | NA |  | NA | H(29/29); L(3/9) | H(37/21); L(5/7) |
| **23** | 2009 | Ma[[23](#_ENREF_23)] | | China | larynx | 40 | NA | 50%(30/10) | cd44-v3 | H(10/20);L(9/1) | H(16/14); L(1/9) | NA | NA | NA | NA | NA |
| **24** | 2011 | Kokko[[24](#_ENREF_24)] | | Finland | oral | 68 | median 120 months | 50%(35/33) | pan-cd44 | NA | NA | NA | NA | NA | H(26/9); L(15/18) | H(29/6); L(26/7) |
|  | 2011 | kokko | | Finland | pharyx | 37 | median 120 months | 50%(23/14) | pan-cd44 | NA | NA | NA | NA | NA | H(19/4); L(4/10) | H(22/1); L(6/8) |
|  | 2011 | Kokko | | Finland | larynx | 30 | median 120 months | 50%(16/14) | pan-cd44 | NA | NA | NA | NA | NA | H(12/4);L(6/8) | H(13/3); L(7/7) |
| **25** | 2011 | Uwa[[25](#_ENREF_25)] | | Japan | pharynx | 40 | median 37 months | 50%(23/17) | pan-cd44 | H(12/11);L(10/7) | H(8/15); L(3/14) | H(9/14);L(1/16) | NA | H(10/7);L(5/18) | NA | NA |
| **26** | 2011 | Yuce[[26](#_ENREF_26)] | | Turkey | larynx | 60 | NA | 25%(29/31) | pan-cd44 | H(5/24);L(3/28) | H(19/10); L(11/20) | NA | NA | NA | NA | NA |
| **27** | 2011 | Lu SM[[27](#_ENREF_27)] | | China | larynx | 66 | NA | cytoplasm staining (18/48) | pan-cd44 | NA | H(13/5);L(17/31) | NA | H(4/14); L(27/21) | NA | NA | NA |
| **28** | 2011 | Zhang[[28](#_ENREF_28)] | | China | oral | 49 | NA | 25%(5/44) | cd44-v6 | NA | H(2/3);L(40/4) | NA | H(5/0); L(23/21) | NA | NA | NA |
| **29** | 2012 | Lindaquist[[29](#_ENREF_29)] | | Sweden | tougue | 62 | median 37months(0-178months ) | 50%(44/18) | pan-cd44 | NA | NA | NA | NA | NA | H(14/30); L(2/16) | H(20/24); L(3/15) |
| **30** | 2013 | steven[[30](#_ENREF_30)] | | USA | oral | 69 | median 32 months (3-88month) | 50%(47/22) | pan-cd44 | H(26/22);L(11/9) | H(17/30);L(10/12) | NA | NA | H(25/22); L(14/8) | NA | NA |

P, positive; N, negative; NA, not available; DFS, disease free survival; OS, overall survival.

**References**

1. Mori S, Nose M, Morikawa H, Sato A, Saito T, et al. (1998) A novel evaluation system of metastatic potential of oral squamous cell carcinoma according to the histopathological and histochemical grading. Oral Oncol 34: 549-557.

2. Ue T, Yokozaki H, Kagai K, Higashikawa K, Yasui W, et al. (1998) Reduced expression of the CD44 variant exons in oral squamous cell carcinoma and its relationship to metastasis. J Oral Pathol Med 27: 197-201.

3. X Song, J Peng, X Cai, F Hou, Lin Q (1999) Expression of CD44v6 protein in laryngeal squamous cell carcinoma. Chinese Journal of Otorhinolaryngology-Skull Base Surgery 5: 4.

4. Masuda M, Kuratomi Y, Shiratsuchi H, Nakashima T, Naonobu K, et al. (2000) Decreased CD44H expression in early-stage tongue carcinoma associates with late nodal metastases following interstitial brachytherapy. Head Neck 22: 662-665.

5. Wang; S, Qiang; Y, Xiao; K, Jiang; S, Chen E (2000) Expression of CD44s and CD44v6 in laryngeal squamous cell carcinoma and their significance. J Clin Otorhinolaryngol (China) 14: 3.

6. X Z Rong, WS Zhi, Ming CX (2001) THE IMMUNOHISTOCHEMICAL EXPRESSION OF CD44s AND CD44v6 IN ORAL SQUAMOUS CELL CARCINOMAS. Journal of Oral and Maxillofacial Surgery 11.

7. Fonseca I, Pereira T, Rosa-Santos J, Soares J (2001) Expression of CD44 isoforms in squamous cell carcinoma of the border of the tongue: A correlation with histological grade, pattern of stromal invasion, and cell differentiation. J Surg Oncol 76: 115-120.

8. Bankfalvi A, Krassort M, Buchwalow IB, Vegh A, Felszeghy E, et al. (2002) Gains and losses of adhesion molecules (CD44, E-cadherin, and beta-catenin) during oral carcinogenesis and tumour progression. J Pathol 198: 343-351.

9. Gonzalez-Moles MA, Bravo M, Ruiz-Avila I, Esteban F, Rodriguez-Archilla A, et al. (2003) Adhesion molecule CD44 as a prognostic factor in tongue cancer. Anticancer Res 23: 5197-5202.

10. SW Zhao, BC Sun, J He, Q Ye, SZ Lin, et al. (2003) The expression of CD44 and its significance in human laryngeal carcinoma. J Clin Otorhinolaryngol(China) 17: 3.

11. Xu YP, Zhao XQ, Sommer K, Moubayed P (2003) Correlation of matrix metalloproteinase-2, -9, tissue inhibitor-1 of matrix metalloproteinase and CD44 variant 6 in head and neck cancer metastasis. J Zhejiang Univ Sci 4: 491-501.

12. Kawano T, Nakamura Y, Yanoma S, Kubota A, Furukawa M, et al. (2004) Expression of E-cadherin, and CD44s and CD44v6 and its association with prognosis in head and neck cancer. Auris Nasus Larynx 31: 35-41.

13. M Wang, L Xing, Zhang C (2004) Study in the Expression of CD44 in Nasopharyngeal Neoplasms and the Clinical Relationship. Tianjin Medical Journal 32: 3.

14. Esteban F, Bravo JJ, Gonzalez-Moles MA, Bravo M, Ruiz-Avila I, et al. (2005) Adhesion molecule CD44 as a prognostic factor in laryngeal cancer. Anticancer Res 25: 1115-1121.

15. XX Qiu, JF Huang, ZX Zhang, P Da, Zhou R (2006) The expression of CD44 and its clinical significance in human laryngeal carcinoma. Medical Journal of Communication 20.

16. Liqun Xie, Lijia Shen, Huiyong Jiang, Liu Q (2006) Tissue microarray assay of CD44v6 and PCNA expression for oral squamous cell carcinomas and precancerous lesions and it sclinical significance. Journal of Jinan University 27: 8.

17. Kosunen A, Pirinen R, Ropponen K, Pukkila M, Kellokoski J, et al. (2007) CD44 expression and its relationship with MMP-9, clinicopathological factors and survival in oral squamous cell carcinoma. Oral Oncol 43: 51-59.

18. Staibano S, Merolla F, Testa D, Iovine R, Mascolo M, et al. (2007) OPN/CD44v6 overexpression in laryngeal dysplasia and correlation with clinical outcome. Br J Cancer 97: 1545-1551.

19. S Huang, W Luo, S Wu, W Wang, Chen X (2007) Expression of PCNA and CD44V6 in laryngeal carcinoma. Journal of Guangdong Medical college 2: 3.

20. J Lu, Y Gao, Y Xiao, Jin D (2008) Expression and clinical significance of CD44v6 in tissues of laryngeal and hypopharyngeal squamous cell carcinoma. Journal of Harbin medical university 42: 5.

21. Gao Y (2009) The expression and its significance of CD44v6 in human laryngeal and hypopharyngeal squamous cell carcinoma. Chinese medical herald 6: 2.

22. X Guo, X Li F, S Feng, X Li, Z Pan, et al. (2009) Expression of gene BRMS1 and CD44v6 protein in supraglottic laryngeal carcinoma and its clinical significance. Journal of clinical Otorhinolaryngology Head and Neck Surgery (China) 23: 6.

23. D Ma, L Yuan, Y Zhao, X Zhou, C Li, et al. (2009) Relationship study between KAI1, CD44v3 expressions and laryngeal carcinoma. Journal of Shandong medical College 31: 4.

24. Kokko LL, Hurme S, Maula SM, Alanen K, Grenman R, et al. (2011) Significance of site-specific prognosis of cancer stem cell marker CD44 in head and neck squamous-cell carcinoma. Oral Oncol 47: 510-516.

25. Uwa N, Kataoka TR, Torii I, Sato A, Nishigami T, et al. (2011) CD44 expression is related to poor prognosis of hypopharyngeal squamous cell carcinoma. Acta Otolaryngol 131: 323-329.

26. Yuce I, Bayram A, Cagli S, Canoz O, Bayram S, et al. (2011) The role of CD44 and matrix metalloproteinase-9 expression in predicting neck metastasis of supraglottic laryngeal carcinoma. Am J Otolaryngol 32: 141-146.

27. Lu S, Tian J, Lv Z, Wang H, Bai X, et al. (2011) The probable role of tumor stem cells for lymph node metastasis in supraglottic carcinoma. Pathol Oncol Res 17: 33-38.

28. R Zhang, LV Fan, S Mu, X Xie, M Zhang, et al. (2011) Expression of CD44v6 in human oral squamous cell carcinoma. Journal of Oral Science Research 27: 4.

29. Lindquist D, Ahrlund-Richter A, Tarjan M, Tot T, Dalianis T (2012) Intense CD44 expression is a negative prognostic factor in tonsillar and base of tongue cancer. Anticancer Res 32: 153-161.

30. Wang SJ, Earle C, Wong G, Bourguignon LY (2013) Role of hyaluronan synthase 2 to promote CD44-dependent oral cavity squamous cell carcinoma progression. Head Neck 35: 511-520.
