# Supplementary material for: Significance of CD44 expression in head and neck cancer: a systemic review and meta-analysis
Source: BMC Cancer. 2014 Jan 13;14:15. doi: 10.1186/1471-2407-14-15 (PMC3893437; doi:10.1186/1471-2407-14-15)
Supplement: Additional file 3 — PRISMA 2009 Flow Diagram. [file 1471-2407-14-15-S3.doc]

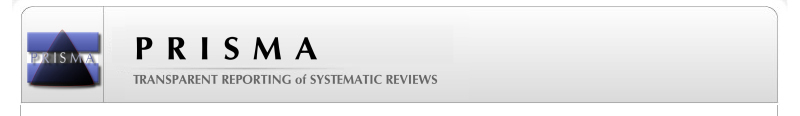
**PRISMA 2009 Flow Diagram**

**Screening**

**Included**

**Eligibility**

**Identification**

Records identified through pubmed, ISI web of Science and CNKI database searching
(n =474)

223 articles retrieved for detailed assessment

Totally 251 Records excluded

218 irrelavant

11 review

22 no desirable outcome

Articles excluded, with reasons:

without clinicopathological and overall survival data

n=139

30 articles included in final analysis

84 articles screened through full text reading

Articles excluded, with reasons:

without detailed clinicopathological or OS data for analysis

(n=24)

n=54
