# Supplementary material for: Significance of CD44 expression in head and neck cancer: a systemic review and meta-analysis
Source: BMC Cancer. 2014 Jan 13;14:15. doi: 10.1186/1471-2407-14-15 (PMC3893437; doi:10.1186/1471-2407-14-15)
Supplement: Additional file 4: Table S2 — Heterogeneity test and publication bias analyses among studies included. [file 1471-2407-14-15-S4.doc]

**Additional file 4:** Table S2 Heterogeneity test and publication bias analyses among studies included

|  |  | T category  (T3,4 vs. T1,2) | | | | N category  (positive vs.negative) | | | | M category  (positive vs.negative) | | | | Grade (grade 3 vs.grade 1,2) | | | | DFS (death vs.survive) | | | | OS 3 years (death vs.survive) | | | | OS 5 years (death vs.survive) | | | |
| --- | --- | --- | --- | --- | --- | --- | --- | --- | --- | --- | --- | --- | --- | --- | --- | --- | --- | --- | --- | --- | --- | --- | --- | --- | --- | --- | --- | --- | --- |
|  |  | Heterogeneity | | Publication  bias | | Heterogeneity | | Publication  bias | | Heterogeneity | | Publication  bias | | Heterogeneity | | Publication  bias | | Heterogeneity | | Publication  bias | | Heterogeneity | | Publication  bias | | Heterogeneity | | Publication  bias | |
|  |  | P^a^ | I^2^(%)^b^ | P^c^ | P^d^ | P^a^ | I^2^(%)^b^ | P^c^ | P^d^ | P^a^ | I^2^(%)^b^ | P^c^ | P^d^ | P^a^ | I^2^(%)^b^ | P^c^ | P^d^ | P^a^ | I^2^(%)^b^ | P^c^ | P^d^ | P^a^ | I^2^(%)^b^ | P^c^ | P^d^ | P^a^ | I^2^(%)^b^ | P^c^ | P^d^ |
| Over all |  | 0.006 | 56.4 | 0.077 | 0.05 | 0 | 71.8 | 0.224 | 0.079 | 0.258 | 25.6 | 1 | 0.911 | 0.003 | 69.8 | 0.548 | 0.256 | 0.001 | 78.3 | 0.462 | 0.976 | 0 | 87.5 | 0.474 | 0.369 | 0 | 83.7 | 0.537 | 0.461 |
| Cancer type |  |  |  |  |  |  |  |  |  |  |  |  |  |  |  |  |  |  |  |  |  |  |  |  |  |  |  |  |  |
| Oral |  | 0.187 | 42.5 | 1 | - | 0.026 | 54.2 | 0.602 | 0.656 | 0.821 | 0 | 1 | - | 0.364 | 0 | 1 | - | 0.168 | 40.7 | 0.308 | 0.522 | 0 | 88.3 | 1 | 0.532 | 0 | 87.5 | 0.462 | 0.545 |
| Larynx |  | 0 | 77.1 | 0.019 | 0.033 | 0.045 | 46.5 | 0.005 | 0 | - | - | - | - | 0.001 | 81.3 | 0.734 | 0.049 | - | - | 0.308 | 0.522 | 0 | 90.7 | 0.806 | 0.901 | 0.027 | 63.5 | 1 | 0.137 |
| pharynx&Larynx |  | 0.002 | 64 | 0.013 | 0.033 | 0 | 81.2 | 0.037 | 0 | 0.002 | 89.1 | 1 | - | 0 | 82.4 | 0.221 | 0.01 | - | 0 | - | - | 0 | 88.1 | 0.76 | 0.454 | 0 | 75.2 | 0.548 | 0.355 |
| Geographic area |  |  |  |  |  |  |  |  |  |  |  |  |  |  |  |  |  |  |  |  |  |  |  |  |  |  |  |  |  |
| Asia | over all | 0.051 | 48.3 | 0.048 | 0.031 | 0 | 79.2 | 0.392 | 0.016 | 0.135 | 50.1 | 1 | 0.974 | 0.001 | 77.3 | 0.707 | 0.238 | 0.009 | 85.5 | 1 | - | 0.005 | 76.5 | 0.308 | 0.096 | 0.005 | 77 | 1 | 0.798 |
|  | oral | - | - |  |  | 0.001 | 85.2 | 1 | 0.549 | - | 0 | - | - | - | - | - | - | - | - | - | - | - | - | 0.308 | 0.096 | - | - | 1 | - |
|  | Larynx | 0.067 | 51.5 | 0.133 | 0.059 | 0.019 | 54.4 | 0.012 | 0 | - | - | - | - | 0.001 | 81.3 | 0.734 | 0.039 | - | - | - | - | 0.093 | 58 | 1 | 0.448 | 0.059 | 64.7 | 1 | 0.584 |
|  | Larynx&pharynx | 0.051 | 48.3 | 0.048 | 0.031 | 0 | 82.4 | 0.1 | 0 | 0.044 | 75.4 | 1 | - | 0 | 82.4 | 0.221 | 0.01 | - | - | - | - | 0.005 | 76.5 | 0.308 | 0.096 | 0.005 | 77 | 1 | 0.798 |
| Europe | over all | 0.156 | 46.2 | 0.296 | 0.056 | 0.048 | 55.2 | 0.707 | 0.137 | - | 0 | - | - | - | - | - | - | 0.04 | 76.3 | 1 | - | 0 | 88.4 | 1 | 0.14 | 0 | 86.9 | 0.386 | 0.154 |
|  | oral | - | - | - | - | 0.521 | 0 | 0.806 | 0.67 | - | 0 | - | - | - | - | - | - | 0.04 | 76.3 | 1 | - | 0 | 88.3 | 1 | 0.532 | 0 | 87.5 | 0.462 | 0.545 |
|  | Larynx | 0.587 | 0 | - | - | - | 0 | - | - | - | - | - | - | - | - | - | - | - | - | 1 | - | 0.005 | 87.3 | 1 | - | 0.674 | 0 | 1 | - |
|  | Larynx&pharynx | 0.587 | 0 | - | - | - | - | - | - | - | - | - | - | - | - | - | - | - | - | 1 | - | 0 | 91.2 | 1 | 0.123 | 0.148 | 47.7 | 0.296 | 0.274 |
| Oral | Pan-CD44 | 0.187 | 42.5 | 1 | - | 0.324 | 11.2 | 0.296 | 0.257 | - | - | 1 | - | 0.346 | 0 | 1 | - | 0.343 | 0 | 1 | - | 0 | 88.3 | 1 | 0.532 | 0 | 89.3 | 0.734 | 0.343 |
|  | CD44-6 | - | - | - | - | 0.124 | 57.7 | 1 | - | - | - | 1 | - | - | - | 1 | - | - | - | 1 | - | - | - | 1 | - | - | - | 0.734 | 0.343 |
| Larynx | Pan-CD44 | 0 | 80.3 | 0.221 | 0.071 | 0.255 | 23.9 | 0.452 | 0.009 | - | - | 1 | - | 0.001 | 81.3 | 0.734 | 0.039 | - | - | 1 | - | 0 | 90.7 | 0.806 | 0.901 | 0.026 | 72.5 | 1 | 0.486 |
|  | CD44-6 | 0.744 | 0 | 1 | - | 0.299 | 18.4 | 0.308 | 0.111 | - | - | 1 | - | - | - | 0.734 | 0.039 | - | - | 1 | - | - | - | 0.806 | 0.901 | 0.531 | 0 | 1 | - |
| Larynx&pharynx | Pan-CD44 | 0.001 | 75.3 | 0.26 | 0.074 | 0.372 | 7.4 | 0.368 | 0.027 | - | - | - | - | 0.001 | 81.3 | 0.734 | 0.039 | - | - | - | - | 0 | 88.9 | 1 | 0.712 | 0.001 | 81.4 | 0.308 | 0.145 |
|  | CD44-6 | 0.501 | 0 | 0.296 | 0.252 | 0 | 80.8 | 0.221 | 0.039 | - | - | - | - | - | - | 0.734 | 0.039 | - | - | 1 | - | - | - | 1 | - | 0.531 | 0 | 1 | - |
| Sample size^a^ | <58.5 | 0 | 83.6 | 0.734 | 0.233 | 0.003 | 67.8 | 0.108 | 0.213 | - | 0 | - | - | 0.1 | 56.5 | 1 | 0.884 | 0 | 87.2 | 0.296 | 0.319 | 0.681 | 0 | 1 | 0.862 | 0 | 86.4 | 0.707 | 0.316 |
|  | ≥58.5 | 0.19 | 28.7 | 0.348 | 0.29 | 0 | 74.9 | 0.553 | 0.268 | 0.337 | 8 | 1 | 0.546 | 0.005 | 76.3 | 0.089 | 0.214 | 0.343 | 0 | 1 | - | 0 | 90.2 | 0.764 | 0.819 | 0 | 85 | 0.707 | 0.83 |
| Follow time(month)^b^ | <68.5 | 0.247 | 23.8 | 0.764 | 0.718 | 0 | 72.8 | 0.474 | 0.498 | 0.374 | 0 | 1 | - | 0.003 | 75.1 | 0.0027 | 0.119 | 0.001 | 78.3 | 0.462 | 0.976 | 0 | 91.1 | 1 | 0.793 | 0 | 87.1 | 1 | 0.948 |
|  | ≥68.5 | 0 | 77.7 | 0.024 | 0.014 | 0 | 73.3 | 0.246 | 0.067 | 0.141 | 53.9 | 1 | - | 0.099 | 63.2 | 1 | - | - | - | 0.462 | 0.976 | 0.514 | 0 | 1 | 0.544 | 0.183 | 38.1 | 0.308 | 0.459 |

a P for heterogeneity within each subgroup.
b Proportion of between-study heterogeneity accounting for total heterogeneity.
c P values of Begg’s test.
d P values of Egger’s test.
