# Supplementary figures and images for: Significance of CD44 expression in head and neck cancer: a systemic review and meta-analysis
Source: BMC Cancer. 2014 Jan 13;14:15. doi: 10.1186/1471-2407-14-15 (PMC3893437; doi:10.1186/1471-2407-14-15)

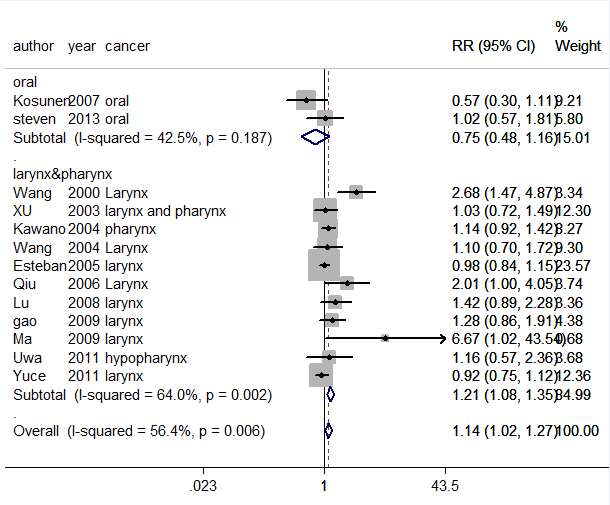

Supplement: Additional file 5: Figure S1 — CD44 expression and T category stratified to oral and pharyngolaryngeal cancer. [file 1471-2407-14-15-S5.tiff]

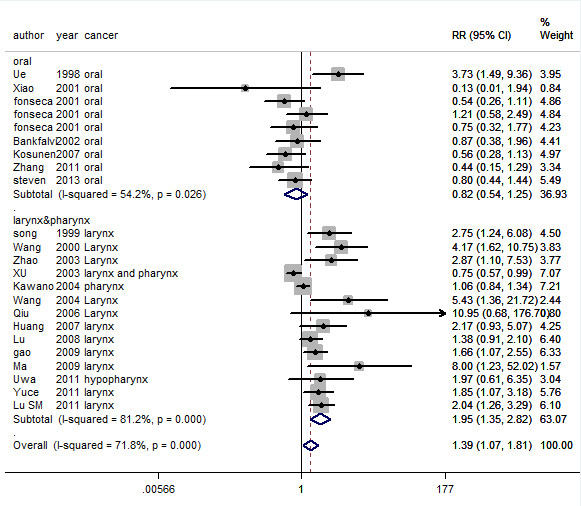

Supplement: Additional file 6: Figure S2 — CD44 expression and N category stratified to oral and pharyngolaryngeal cancer. [file 1471-2407-14-15-S6.tiff]

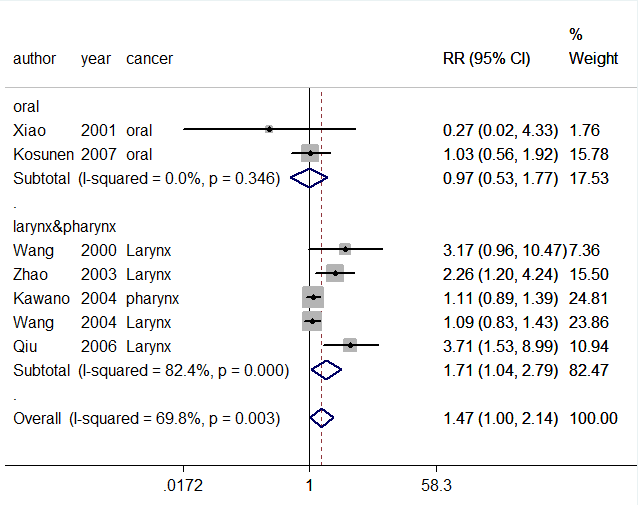

Supplement: Additional file 7: Figure S3 — CD44 expression and tumor grade stratified to oral and pharyngolaryngeal cancer. [file 1471-2407-14-15-S7.tiff]

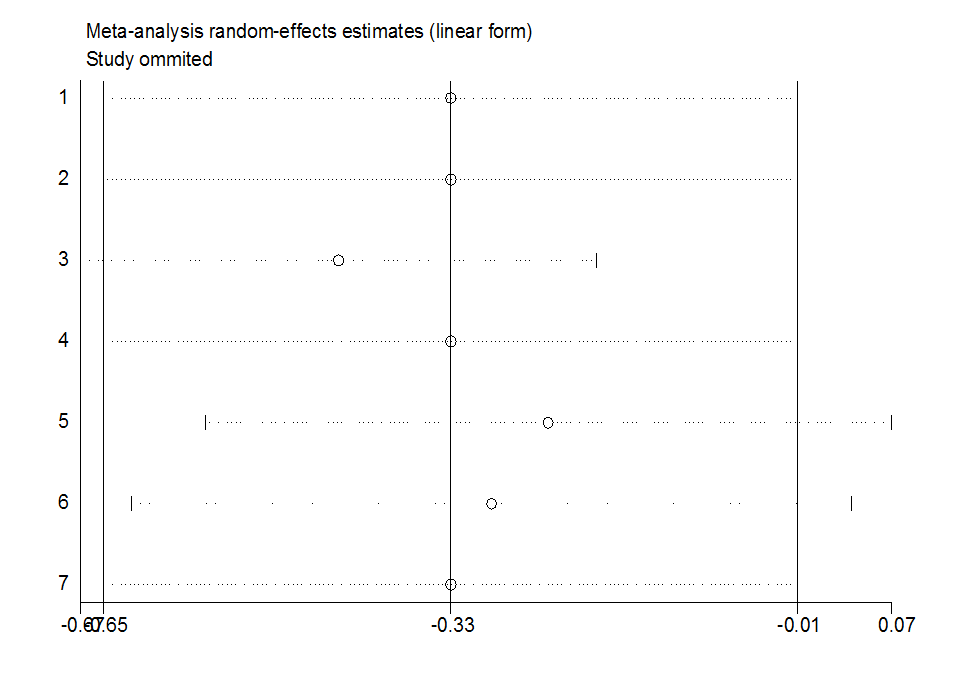

Supplement: Additional file 8: Figure S4 — Sensitivity analysis of 5-year OS rate. [file 1471-2407-14-15-S8.tiff]
